# Writing Prescriptions

**Published:** 1869-12

**Authors:** 


***Writing Prescriptions.***

IN no particular is perfect exactitude and care more requisite than in writing prescriptions. If the proposed action is taken by the American Medical Association, forbidding, under pains and penalties, physicians from dispensing medicines when there are druggists "handy," these will become even more indispensable. We are no admirers of polypharmacy, and yet admit the advantage of suitable "adjuvants and corrigents" to the essential ingredient of a formula. It is clearly desirable that the several members should be neither physiologically nor chemically incompatible.

It is proper that the names of the ingredients should be written in officinal form, and with the least possible abbreviation. The scientific name is universal, the popular (or "English") name is local and variable.

Directions should be written in the vernacular. The whole should be "concise, precise, and decisive." Thereafter the responsibility rests upon the druggist. It is too much to ask of the druggist to read good or bad Latin sentences. It is not too much to demand of him knowledge of the authorized and standard scientific names of drugs.

But there are several things which puzzle us daily. For instance, why do well-educated and competent physicians, prescribing (just now) the *Bromide of Potassium*, always write it, in defiance of the nomenclature, *Bromide of Potash?* The same vice was seen a little while ago when Iodide of Potassium was the favorite panacea—it was always the Iodide of *Potash.*

Nothing is more common than to see the name written half Latin and half English. Or, again, it is written in the nominative instead of the genitive case.

Such blunders make the profession a laughing stock to even tolerably educated men.

Again: the punctuation is almost invariably defective. A curious example of this is seen in the, generally, very correct text of Elmer's Hand-Book for 1870. In giving several illustrations of prescriptions, they put a *period* after each article named. It is unnecessary to say that a semicolon is the proper punctuation. Again: they assume, contrary to the historical fact, that the character ℞ is an abbreviation for — *Recipe* (*take thou*), whereas it is merely the arbitrary sign of a medical prescription, and was anciently a hieroglyphic of invocation. The governing verb is *Misce*, and this requires the genitive. In either case the periods are wrong, as the sentence is not completed. They also give the druggist his directions in Latin (p. 113), which practically is dangerous.

Another trouble is illustrated in the following old-fashioned prescription:

| | | |
|---|---|---|
| ℞ | Mass. ex Hydrarg., - - - - | } aa gr. xij; |
| | Pulv. Aloes Socot., - - - - | |
| | Pulv. Ipecac., - - - - - | gr. ij; |
| M. | | |

One druggist will put this up with *six* grains each of the blue mass and aloes, and another will put in *twelve* grains of each. The introduction of the brace ordinarily employed to assure the druggist that there has been no accidental omission of a quantity (more possible when three or more medicines are used) will perplex him, with chances that he will get it wrong, whichever way is desired.

A little knowledge is a dangerous thing, and unfortunately our friends, the apothecaries, illustrate this too often for our personal happiness. Substitution of similar medicines (tannin for gallic acid, for instance) is among the least of the liberties they take with our formulæ. They should be held responsible for dispensing the prescription exactly as it is written, unless there is clearly an error of a dangerous nature, when it is their business at once to notify the author.

SPEAKING of druggists, reminds us that the Chicago College of Pharmacy at a recent meeting (Nov. 17), took the following action:

WHEREAS, A Convention has been called for the revision of the U. S. Pharmacopœia to meet in Washington, in May, 1870, and it being desirable to make the proposed revision as complete and perfect as possible, and

WHEREAS, The interests of the Medical profession are identical with our own, in this, that the preparations and processes of the Pharmacopœia should be made to correspond with the ascertained wants of the profession and to the advancement in science during the past decade, and

WHEREAS, Our united efforts will enable us to accomplish more successfully the desired results, therefore be it

*Resolved*, That the members of the Medical profession and the Druggists of Chicago be respectfully invited to attend the Pharmacal Meetings of this College and participate in the discussions upon the next decennial revision of the U. S. Pharmacopœia.

The Pharmacal Meetings will be held on Wednesday evening of each week, commencing at 8 o'clock, in the rooms of the College, Room 12, 77 Dearborn street.

### *BACK NUMBERS.*

THOSE subscribers who have written for back numbers of the JOURNAL will be supplied soon. Temporary *indisposition* of our previous publisher (J. W. H.) causes a little delay, which certain proceedings I have instituted will speedily abate. J. ADAMS ALLEN.

